# Mentalizing Bodies: Explicit Mentalizing Without Words in Psychotherapy

**DOI:** 10.3389/fpsyg.2021.577702

**Published:** 2021-06-07

**Authors:** Thomas Wiben Jensen, Stine Steen Høgenhaug, Morten Kjølbye, Marie Skaalum Bloch

**Affiliations:** ^1^Department of Language and Communication, University of Southern Denmark, Odense, Denmark; ^2^Outpatient Clinic of Anxiety Disorders and Personality Disorders, Psychiatrist Hospital, Aalborg University Hospital, Brønderslev, Denmark

**Keywords:** mentalization, social interaction, psychotherapy, embodied cognition, re-enactment, affordance

## Abstract

**Introduction:** Mentalization concerns the human ability to understand the actions of others (and oneself) in terms of intentional mental states. Theoretically, the notion has been described *via* the poles of automatic, non-verbal implicit mentalization as opposed to conscious and verbal explicit mentalization. In this article, we challenge this standard distinction by examining examples from psychotherapy. We argue that explicit mentalization can also be carried out *via* embodied non-verbal actions.

**Method:** Four cases of real-life interaction from psychotherapy sessions are analyzed from the qualitative perspective of embodied cognition and multimodal interaction analysis. The analyses are based on video data transformed into transcriptions and anonymized drawings from a larger cognitive ethnography study conducted at a psychiatric hospital in Denmark.

**Results:** The analyses demonstrate the gradual development from predominantly implicit mentalizing to predominantly explicit mentalizing. In the latter part of the examples, the mentalizing activity is initiated by the therapist on an embodied level but in an enlarged and complex manner indicating a higher level of awareness, imagination, and reflection. Thus, the standard assumption of explicit mentalization as contingent on verbal language is challenged, since it is demonstrated how processes of explicit mentalization can take place on an embodied level without the use of words.

**Conclusion:** Based on real-life data, the study demonstrates that online processes of implicit and explicit mentalization are gradual and interwoven with embodied dynamics in real-life interaction. Thus, the analyses establish a window into how mentalization is carried out by psychotherapists through interaction, which testifies to the importance of embodied non-verbal behavior in psychotherapy. Further, informed by the notion of affordance-space, the study points to alternative ways of conceptualizing the intertwined nature of bodies and environment in relation to conveying more complex understandings of other people.

## Introduction

The notion of mentalization designates the capacity to understand each other as mindful beings driven by emotions, wishes, needs, desires, and so forth and to be aware of these motivating factors when we engage in social interaction (Fonagy et al., 2002; Bateman and Fonagy, 2006). The concept of mentalization was originally coined and developed by Fonagy and colleagues in relation to their work with patients with borderline personality disorders (Allen et al., 2003; Bateman and Fonagy, 2012). Today, mentalization is widely used as a clinical framework for treating a number of disorders, such as post-traumatic stress disorder, eating disorders, and depression (Bateman and Fonagy, 2010). Furthermore, mentalization is now also more broadly used as a general concept aimed at an important intersubjective aspect of human understanding, which is used in many different contexts such as social work in schools (Twemlow and Fonagy, 2006), in health communication, and in organizational practice (Fonagy, 2006). A defining characteristic of the concept of mentalization is the way it is structured around four different dimensions, each defined in virtue of two polarities with a gradual transition between them: self–other, affective–cognitive, internal–external, and explicit–implicit.

In this article, the authors present a qualitative study with a specific focus on empirically investigating the relationship between *implicit* and *explicit* mentalization from real-life data from psychotherapy. Accordingly, the study provides in-depth analyses of actual practices of mentalizing in social interaction, as opposed to *post-hoc* interviews with patients or therapists or experimental data from a clinical setting. This qualitative vantage point allows for more detailed investigations of some of the basic assumptions within the literature on mentalization, in particular, the distinction between explicit and implicit mentalizing. In a recent publication, Fonagy and Bateman (2019) describe this dimension of mentalizing in the following way:

The most fundamental dimension to mentalizing is the spectrum between automatic (or implicit) and controlled (or explicit) mentalizing. Controlled mentalizing reflects a serial and relatively slow process, which is typically verbal and demands reflection, attention, awareness, intention, and effort. The opposite pole of this dimension, automatic mentalizing, involves much faster processing, tends to be reflexive, and requires little or no attention, intention, awareness, or effort. (p. 8).

In the literature on mentalization, implicit mentalizing, on the one hand, is described as the most basic and widespread form of mentalizing (Fonagy et al., 2002; Bateman and Fonagy, 2006; Fonagy and Luyten, 2009). Implicit mentalization is typically manifested in human interaction *via* bodily actions, such as facial expressions, eye contact, head nods, gesture, and in-breaths. These bodily cues are crucial in supporting alignment and a shared understanding between interlocutors, even though they often occur without much conscious effort or awareness (Shai and Belsky, 2011a; Liljenfors and Lundhl, 2014; Davidsen and Fosgerau, 2015; Fosgerau et al., 2017). Explicit mentalization, on the other hand, is described as distinctively different from these non-verbal behaviors since it is manifested *via* actions that are “interpreted, conscious, verbal, and reflective; it is a slow process that necessitates awareness and effort” (Shai and Belsky, 2011b, p. 188). In short, it is assumed that since implicit mentalizing behavior is non-verbal, explicit mentalizing, by definition, relies on verbal actions allowing for more deliberate and conscious reflection.

In this article, we challenge this basic assumption in mentalization theory by investigating examples of mentalizing actions from psychotherapy that, to varying degrees, require attention, awareness, effort, and conscious actions without involving verbal language. In doing so, we undertake an embodied perspective on mentalization informed by a vantage point in phenomenology and embodied cognition (Merleau-Ponty, 1962; Thompson, 2007; Colombetti, 2014; Gallagher, 2017). This perspective emphasizes both the essential role of the body and the crucial role of the environment in the cognitive abilities. These theoretical frameworks are important for this study since they support the idea of conscious reflective mentalizing as unfolding within embodied actions rather than relying on language. The contemporary view on embodied cognition stresses the vital role of embodied actions in the ways in which we think, feel, and reason both about ourselves and in relation to other people. Thus, another central objective of the article is to offer an alternative way of thinking about the role of the bodies in conveying more complex understandings of other people.

In sum, in this qualitative study, the authors explore the poles of implicit and explicit mentalization, and the space between the poles, by analyzing four different examples from psychotherapy that (1) exemplify both the range and interrelated nature of the elements involved in implicit and explicit mentalization and (2) challenge the standard assumption of explicit mentalization as primarily based on verbal language. The overall argument is that explicit mentalization can also be part of a non-verbal embodied behavior.

Following from this, we ask the questions:

- How does the line between implicit and explicit mentalizing unfold in practice in real-life psychotherapy data from the perspective of embodied interaction?
- How do implicit and explicit mentalizing interact in the ongoing flow of interaction, and how can explicit mentalizing take place on an embodied level without the use of verbal language?

The examples are based on video recordings of therapy sessions making it possible to examine mentalizing behavior embedded in the structures of social interaction. The selection of examples illustrates the gradual development from predominantly implicit mentalizing to predominantly explicit mentalizing. In the latter part of the examples, the mentalizing activity is initiated by the therapists on an embodied, non-verbal level, but in a marked, enlarged, and complex manner that suggests the involvement of a conscious reflective level.

The empirical ambition of the study is to provide qualitative in-depth analyses based on authentic data in relation to mentalization, specifically in relation to the gradual transition between implicit and explicit mentalization. In this regard, this study can be seen in conjunction with a recent trend in mentalization studies to work with real-life data from the perspective of different types of interaction analysis (Davidsen and Fosgerau, 2015; Fosgerau et al., 2017; Keselman et al., 2018; Samuelsson and Rosberg, 2018; Shaw et al., 2019).

## Theoretical Framework

### Mentalization

Mentalization refers to the human capacity to “*hold mind in mind*” (Allen, 2006, p. 3), that is, to be able to pay attention to and interpret the intentions that drive our actions and behaviors. It addresses the capacity to be aware of the thoughts and the feelings of other people, as well as those of our own, and the ways in which we, implicitly and explicitly, display or communicate this awareness. Thus, the locus of mentalization is the unique human capacity for developing an awareness of the actions of ourselves and others on a meta-level in the sense of being able to imagine and interpret what lies behind the mere physical manifestations of human actions (Fonagy and Luyten, 2009; Liljenfors and Lundhl, 2014; Fonagy and Bateman, 2019). In this regard, the concept of mentalization interfaces with other concepts such as metacognition, social cognition, and empathy (Choi-Kain and Gunderson, 2008; Davidsen and Fosgerau, 2015; Fosgerau et al., 2017). According to mentalization theory, the ability to mentalize is developed and rooted in early childhood experiences. Research shows that the capacity of the parent to consider and treat the child as a psychological agent motivated by mental states is associated with infant attachment security, so that the quality of the relationship between the child and its caretakers impact profoundly the rate of development and the future competence of the child in mentalizing in general (Fonagy et al., 2002; Shai and Belsky, 2011a).

In a clinical context, the concept of mentalization was used by Fonagy and Bateman (Fonagy, 1991; Fonagy and Target, 1996; Bateman and Fonagy, 2006) in relation to treating patients with borderline personality disorders (BPD). Following from this, the notion of mentalization has led to the psychodynamic treatment program *Mentalization-Based Treatment* (MBT). MBT was designed for people with BPD who suffered from disorganized attachment, thereby failing to develop a sustainable capacity for mentalization. Therefore, the aim of MBT treatment is related to increasing and consolidating the ability to mentalize, so that patients can improve their affect regulation and hence strengthen their interpersonal relationships. More recently, the notion of *epistemic trust* (Fonagy and Allison, 2014) has been introduced to underline the importance of the “mentalizing therapist” as a common factor in psychotherapy. Epistemic trust refers to the level of trust an individual dares to offer in relation to social learning in an attachment context. In the case of relational childhood trauma, traumatized individuals may protect themselves by the defensive exclusion of information in social situations and, hereby, developing epistemic mistrust (Knox, 2016). In this sense, the primary focus for the therapist is to (re)establish a trustful relationship together with the patient to the social world as a learning environment.

### Dimensions of Mentalization

As stated in the introduction, mentalization is a multifaceted phenomenon building on four different dimensions, each related to two different poles that are supposed to rely on distinct neural systems (Sapute and Lieberman, 2006). Several neuroscientific studies have examined the neural grounding for different types of pathological behaviors such as autism in relation to the poles inherent in the different dimensions of mentalization (Jung, 2014; Crespi et al., 2016; Carver et al., 2017).

One dimension is *self-other*, which refers to the fact that mentalizing is considered both an interpersonal and an intrapersonal phenomenon, in the sense that the mentalizing process can be directed at one's own thoughts and feelings as well as at the underlying thoughts and feelings of others. Another related dimension is *internal-external*, which designates the distinction between having a focus on internal thoughts and feelings as opposed to having a focus on external behavior or features that point back to underlying thoughts and feelings. Next, there is the dimension of *cognitive-affective*, which concerns the fundamental distinction between reflective reasoning and thinking and a more basic emotional understanding and attunement. It is important to stress that the mentalizing process is not a fixed property, as it can vary both in relation to the context and the interpersonal relation as well as in relation to the different dimensions (Fonagy and Luyten, 2009; Fonagy and Bateman, 2019). As such, one person can have average or good mentalizing abilities in one area and limitations or impairments in another area (Fonagy and Luyten, 2009).

### Marked Mirroring in the Intersection Between Implicit and Explicit Mentalization

The dimension of *implicit and explicit mentalizing* is typically understood *via* an additional set of distinctions, such as non-verbal (embodied) vs. verbal, non-conscious vs. conscious, reflective vs. unreflective (automatic), and slow vs. fast. There is general agreement that implicit mentalizing is fast, automatic, and non-verbal while explicit mentalization relies on words and reflective conscious awareness (Fonagy and Bateman, 2019). However, in descriptions of so-called *marked mirroring* and affect regulation between the parents and the infants, the distinctions between implicit and explicit mentalizing are more blurred (Fonagy et al., 2002). Marked mirroring concerns the ways in which parents mirror the actions and facial expressions of infants in a way that is both congruent and attuned while also being marked in a way that indicates an affect belonging to the infant, not the parents' own affect. This specific aspect of mentalization concerns the *imaginative quality* of mentalization, that is, the way mentalizing “is positioned within the interpersonal ‘workhorse' of the social imagination” (Fonagy and Bateman, 2019, p. 4). These imaginative behaviors are typical and vital for both playful and comforting episodes between the parent and the child. According to Fonagy et al. (2002), this marking can only be accomplished “by producing an exaggerated version of the realistic emotion expression of the parent, similarly to the marked ‘as-if' manner of emotion display that is characteristically produced in pretend play” (pp. 177–178). As noted by Liljenfors and Lundhl (2014, p. 43), it is important to “mark” the facial expressions for the child to be able to “distinguish the mirrored expression from the parent's own emotional expression—or, in other words, without the ‘marking,' the affects that are expressed would not be perceived by the child as representing the child's own affects.” This suggests a rather complex type of mentalizing activity in which the parent must be able to display affect and emotions belonging to both themselves and the child, requiring some level of conscious reflection and affective imagination. Still, this is an embodied process emerging from the ongoing bodily dynamics and displayed without the use of words, which suggests that the distinction between implicit and explicit mentalizing is less clear-cut than it is often portrayed in the literature.

It is this specific quality of mentalization that is explored in the latter part of the analyses from the perspective of embodied cognition and studies of re-enactment.

### Embodied Cognition and the Phenomenal Body

The core tenet in the notion of embodied cognition is the assumption that the cognitive capacities are fundamentally shaped by the bodily functions. That is, the properties of the human body constrain the concepts that human beings acquire and entertain (Shapiro, 2011). Thus, the workings of the mind are part of the workings of the body from the very start. In this way, such psychological concepts as memory, perception, calculation, and language comprehension are never to be understood as purely mental and abstract categories separated from the bodies. Instead, they are all rooted in bodily experiences and various sensorimotor capacities. In contemporary terminology, this view of the body is often termed as *embodiment* (Gibbs, 2005; Johnson, 2007; Shapiro, 2011). In a historical perspective, this view of the meaning and importance of bodily experiences owes a great deal to the phenomenological tradition, in particular the work of Merleau-Ponty. According to Merleau-Ponty (1962); the human body needs to be conceptualized in terms of both the objective biological body (körper) as a physiological entity on the one hand and the body as an experiential quality (leib) on the other. The latter denotes the *phenomenal body* referring to one's body as it is experienced by the same person. It is this dimension of the bodies that is at the heart of embodiment in the sense that we first and foremost experience our bodies not as a physiological objective reality but rather as a potential for action *via* movement and phenomenal sensitivity. Furthermore, a recent tendency in the field of embodied cognition is inspired by distributed, enactivist, and ecological developments in the field of cognitive science (Thompson, 2007; Hutchins, 2014; Gallagher, 2017), making it possible to claim that the phenomenon we call cognition emerges from processes that are distributed across the brain, the body, and the environment. This development in cognitive science has been described with different terms, such as distributed or embodied, enacted, embedded, and extended (in short 4E cognition) (Menary, 2010). As such, the way the authors use “embodied” in this article is meant to be an umbrella term to some extent encompassing all the different E's. This also implies that the authors, for reasons of space, do not go into discussions on the subtle internal differences between these new approaches while instead focusing on the commonalities that, seen together, set them apart from a more standard in-the-head view on cognition^1^. A shared assumption across the different new approaches is that cognition cannot solely be understood in terms of processes taking place in the head (or in the brain). Thus, even though cognition is clearly dependent on neural activation in the brain, it cannot adequately be understood as an internal process. The key to this new understanding of the notion of “the mind” is to avoid treating percepts, concepts, propositions, and thoughts as quasi-objects (mental entities or abstract structures) while instead seeing them “as patterns of experiential interaction. They are aspects or dimensions or structures of the patterns of organism-environment coupling (or integrated interaction) that constitute experience” (Johnson, 2007 p. 117).

Thus, embodied cognition is bound to action, in the sense that cognition is more than an internal precondition for action. It is not only to be seen as an inner mental architecture that underlines the way we are able to navigate in the world but rather, cognition is that navigation itself. In other words, cognition is understood as the active sensemaking of a living agent that navigates and explores its world in movement, perception, and action. In this way, cognition is no longer understood in terms of an internal, and universally structured, schema but rather as a coupling between the bodies and their environment. This dimension of embodied cognition is of particular interest for the exploration of embodied mentalizing on both an implicit and explicit level that, by definition, involves an embodied intersubjective engagement with the affordances, physical as well as social, of the environment. Imagining what lies behind the actions of the interlocutors, and conveying this understanding, is not a pure “mental act” but rather an aspect of the “patterns of experiential interaction” (Johnson, 2007) in which we think and feel by engaging with the interpersonal environment.

In relation to social interaction, a concrete manifestation of this way of thinking about this embodied interpersonal engagement can be found in the notion of *re-enactment*, which we will briefly introduce in the next section.

### Re-Enactment

The term *re-enactment* addresses the ways in which people engage in social interaction that convey or perform the previous situations during the ongoing flow of conversation. The notion of re-enactment is closely related to, and builds on, *reported speech*, which has been investigated within both literary studies and linguistics (Bakhtin, 1981; Semino and Short, 2004; Prior et al., 2006), as well as *footing* studied within micro-sociology (Goffman, 1979). In both cases, the focus is on how participants, in reporting a previous event, shift perspective and convey that they are “now speaking as someone else” (Goffman, 1979). This can be carried out in a number of ways, most often involving the use of direct speech. As implied in the term re-enactment, “reporting” clearly involves more than just repeating the words from a previous speech event; it is also a way of re-doing or re-playing what took place and, in this way, it involves a strong aspect of acting or performing the action, not just reporting it.

A crucial aspect of re-enactment is the way that the reported action is embodied by participants in the here-and-now by use of gesture, facial expressions, posture, tone of voice, etc. (Streeck, 2009; Jensen, 2014; Jensen and Pedersen, 2016; Goodwin, 2018). In the analytical section, a particular way of performing re-enactment is presented. It is one that is performed by the therapist but carried out without the use of words. Instead, the re-enactments rest on a number of embodied resources such as posture, facial expression, and breathing. Thus, it is in fact not a case of speaking as someone else but instead of *acting* as someone else.

To sum up, the framework of embodied cognition, as well as the notion of re-enactment, involves a perspective on embodied action as much more than just a type of “automatic behavior.” Rather, the bodies are mindful organisms, and embodied action is how we think from the very start. This view builds on the phenomenological tradition of the phenomenal body as the primary source of experience for intersubjective relations. Thinking, reflection, and affect are not disembodied and purely abstract processes. Instead, they are processes that unfold over time and take place in concrete situations, which involve felt experience and a bodily sense of the environment including other people. This perspective will be brought to play in relation to the distinction between implicit and explicit mentalization in the analytical section.

## Data and Method

### Dataset, Ethics, and Methodology

The dataset used in this article comes from a large cognitive, ethnographic study conducted at a Danish psychiatric hospital. The dataset consists of video recordings of authentic therapeutic conversations between therapists and patients diagnosed with social anxiety disorders and/or personality disorders. Altogether, 26 patients were recruited for the study and all sessions of each patient (pending from 6 to 50 sessions per patient) were recorded. The video recording was carried out using two stationary cameras that were placed so as to interfere as little as possible with the therapy. This study was reported to the Southern Danish Regional Committee on Health Research Ethics and the Legal office of the University, in which the project is registered. All patients and therapists have given their written consent. This article uses anonymized versions of the data translated from Danish to English. Written informed consent was obtained from the participants for publication of their cases as well as the accompanying drawings. The drawings were made by a professional illustrator and were completely anonymized; still, due to their level of detail, they allow for a detailed impression of the facial, postural, and gestural actions *in situ* of both the therapist and the patient. The use of drawings has the advantage of giving more direct access to the embodied dynamics of the interaction compared to only a textual transcription.

The interactional method is based on multimodal interaction analysis (MMIA) (Goodwin, 2018). This combines a basic transcription of words; notations of basic prosodic features, such as pitch, volume, speed, intonation, and tone of voice (e.g., smiling or crying voice); and drawings of the interlocutors. MMIA is devised to investigate social interaction as a whole-bodied activity embedded in a physical and social environment. Central to the method is the assumption that verbal and bodily non-verbal dimensions of language are equally important dimensions of language use. This means that MMIA considers the full array of situated embodied actions, including gesture, gaze, facial expression, posture, and head movement, in concordance with verbal utterances. Through MMIA, it is thus possible to investigate language use as an embodied activity connected to affect and emotion in and through embodied actions.

### Analytical Procedure and Case Selection

This paper is a qualitative study of different types of mentalizing actions in relation to the gradual distinction between implicit and explicit mentalization in real-life interaction. To obtain a set of examples from the dataset, the first author collaborated with three of the therapists connected to the project who are also co-authors of the study. Two of them also function as therapists in the cases presented below. All the therapists work with MBT as their primary therapeutic method, and they all have a strong interest in the embodied aspects of mentalization. The examples presented in the analysis are all selected to exemplify the general assumption in the field that the difference between implicit and explicit mentalization exists on a continuum covering implicit, partly implicit, partly explicit, and explicit types of mentalizing activities. To gather relevant examples in relation to this specific dimension of mentalization, the following criteria for selection were defined: (1) the case should involve a situation in which a visible or audible embodied action from the therapist directed at the patient can be detected; (2) the embodied actions should, in varying degrees, function as intelligible part of the interaction independently of verbal actions; and (3) the embodied actions should, to varying degrees, function as mentalizing activity in the sense that they address the mental state of the patients. Ten potential examples were gathered, and the collection was narrowed down to four cases that represent the gradual transition from implicit to explicit mentalization, in the sense that the first examples primarily involve implicit mentalizing while the latter examples are predominantly explicit. The selections, as well as the analyses, are conducted from the perspective of embodied cognition in relation to mentalizing activities. Further, the defining criteria for treating the latter part of the examples as involving explicit mentalization was based on the degree of conscious reflection and imaginative awareness as part of the embodied actions.

## Analyses and Results

### Therapeutic Background for the First Case

The first example involves a male patient (P) in his mid-thirties and a female therapist (T). The patient is suffering from generalized anxiety disorder and panic attacks, and he has previously had problems with eating disorders and drug abuse. During therapy, P has revealed for the first time that as a child he was abused by a teacher. He has never told anyone about this before and he still becomes overwhelmed with guilt and shame when talking about the abuse. Today, P lives in a homosexual relationship and he has just reported to T that together with his husband he wants to adopt a child. However, he now worries that telling about the abuse in therapy might influence the chances of the couple to adopt a child. Thus, in the first part of the sequence (line 1), P conveys his concern that being open about the abuse in his childhood will not only “ruin that chance” of adopting a child but also possibly destroy the relationship with his husband. The therapist then attempts to mirror and display an understanding of this concern.

### Analysis of Example 1

**Danish original version**

1. P: faktisk ehm og der føler jeg lidt jeg nærmest sådan har kunne ødelægge den chance nu
2. [°°hvis man kan sige det sådan°°]
3. T: [.hhh::::::: ] o(hh)kay:: hmm::
4. P: faktisk så det var derfor jeg egentlig aldrig ville have sagt noget herude øh fordi omvendt
5. altså ja
6. T: blev du bange for konsekvenserne for (barnet?)
7. P: jeg blev glad dengang da du sagde til at starte
8. med at fordi at je- der bragte jeg det faktisk allerede på banen hvor du sagde at øh
9. (1:5) at du så du bare kun som noget positivt at øh altså-
10. T: at man arbejder med tingene
11. P: ja at jeg gik i behandling og fik det bedre

**English translation**

1. P: actually ehm and in that respect I feel a little bit that I have almost ruined that chance by now
2. [°°if you can put it like that°°]
3. T: [.hhh::::::: ] o(hh)kay:: hmm::
4. P: actually that's why I in fact never would have said anything out here eh because reversely well
5. yeah
6. T: you became afraid of the consequences [for the child]
7. P: [I was happy] back then when you said to start with that
8. because I actually I already brought it up when you said that eh (1:5) that you only saw it as
9. something positive at eh well I
10. T: that you work with things
11. P: yes that I went into treatment and got better

**Figure 1:**
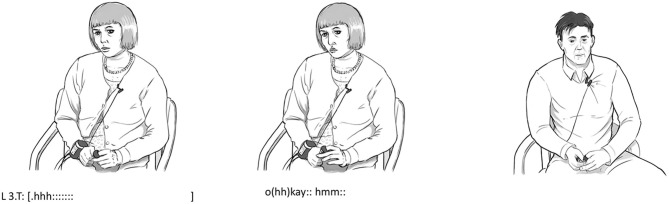
Bodily actions in relation to example 1.

In line 3, after P has just revealed his concern about ruining his chance for adoption (see Figure 1), the therapist makes a deep in-and-outbreath with a slightly prolonged sound that leads into the following “okay.” This clearly audible action is accompanied by the therapist raising her upper body and leaning slightly backward as well as opening her eyes more widely with a concerned look on her face (see Figure 1). These combined bodily actions are carried out in overlap with the remains of the turn of the patient in line 2, which makes it clear that embodied actions of T function as an immediate response to the revelation of T in line 1. In this way, the embodied actions of T display both a slight surprise as well an acknowledgment of the information given in line 1 as being new to the therapist (indicated by the widening of her eyes as well as the sigh itself). Furthermore, the sigh is displayed in such a way that it appears as expressing an emotional stance of sympathy and understanding toward the situation of the patient (appearing right after the utterance of concern of the patient). As such, these embodied non-verbal actions might, on the face of it, be a possible candidate for an embodied type of explicit mentalizing, since they form a visible and audible action directed at the state of the patient. However, the sigh is tightly intertwined with the verbal action. The outbreath leads into the verbal articulation of “okay” and as such, the sigh is not marked as an independent action. Rather, the non-verbal actions (the “okay” is also immediately followed by a “hmm”) are functional parts of the overall verbal action of mirroring and expressing sympathy for the patient, but not as independent actions.

The unmarked and immediate nature of these embodied actions seems to be more in line with the fast, intuitive, and non-conscious character of implicit mentalizing. The in-and-out breath is audible but it does not stand alone, since the last part of it is intertwined with the articulation of the verbal action. Still, it is interesting to notice that this initial implicit act of mentalizing is afterward followed by a verbal and explicit form of mentalizing. A few seconds later, in line 6, the therapist takes the turn and verbalizes how the patient might feel right now: “you became afraid of the consequences.” This explicit shift in perspective seems to have a positive effect on the patient since the patient afterward presents an alternative view focusing on the positive side of going to therapy leading to how he “got better.” To sum up, the outline in the first example seems to fit the description in the literature of implicit mentalizing as non-verbal and largely automatic vs. explicit mentalizing based on verbal actions and reflections.

In the next example, however, the embodied actions seem to have a more independent character.

### Therapeutic Background for the Second Case

The second example involves a 40-year-old female patient and the same female therapist in example 1. The patient has been diagnosed with antisocial personality disorder and paranoid personality traits. A recurrent theme in the therapy sessions is the way the patient protects herself from others, either by means of aggressive behavior or by shutting her emotions down so as not to feel weak. The background for the discussion in the extract is the illness of the mother of the patient. Her mother is sick with terminal cancer and the patient is experiencing emotions of sadness and concern, which the patient usually regards as forbidden emotions making her vulnerable to attacks. The patient therefore reacts by forming a self-protecting strategy of suppressing emotions and by scolding herself in a condescending tone. Previously, this strategy has proved helpful, because it has enabled her to escape from vulnerability and assaults, but now, the therapist attempts to present a new perspective of these emotions as normal and necessary. Right before the sequence, the patient has stated that she is somewhat like a man: tough, strong, and never vulnerable.

### Analysis of Example 2

**Danish original version**

1. P: tører øjnene og så.sh sådan mander mig op ik og så [går jeg videre]
2. T: [du bruger ordet] man ja
3. du siger mande mig op det er jo også noget der kommer fra mand
4. P: ja
5. P: ja (0.5) jamen det er rigtig nok det gør jeg
6. T: men selv mænd bliver jo kede af det når deres mor er ved at dø
7. P: °ja°
8. T: og det er ikke sikkert hun er det men hun er i hvert fald på en eller anden måde lidt i livsfare ik
9. P: ja
10. T: det kan jeg virkelig godt forstår du bliver ked af
11. P: ja
12. T: (2:0).hhh hhhh::
13. P: men jeg har det meget ambivalent med fordi jeg tænker jo meget på det her i øjeblikket
14. ik med at hun risikerer jo at dø og hvad så

**English translation**

1. P: dry your eyes and then like man up right and then [I move on]
2. T: [you use the] word man yes you say man up
3. and that is also something that comes from man
4. P: yes
5. P: yes (0.5) well that is true enough I do that
6. T: but even men get sad when their mother is about to die
7. P: °yes°
8. T: and it is not certain that she is but she is somehow a bit in mortal danger right
9. P: yes
10. T: this I really do understand that it makes you sad
11. P: yes
12. T: (2:0).hhh hhhh::
13. P: but I feel very ambivalent in relation to this because I think a lot about this at the moment right
14. her risking to die and then what

**Figure 2:**
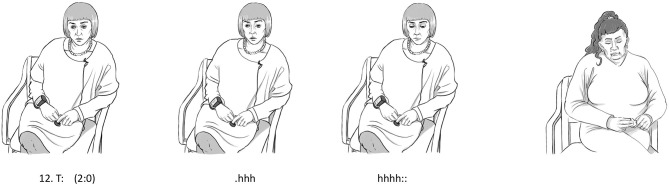
Bodily actions in relation to example 2.

In line 1, the patient uses the conventional expressions “dry your eyes” and “man up” in relation to managing her emotional state regarding the severe illness of her mother. The therapist immediately picks up on the use of the expression “man up.” In lines 2–3, the therapist draws attention to the wordings by remarking on the gender connotations in these expressions in a lighter tone of voice while smiling, indicating a playful dimension in the talk despite the severity of the topic. The patient seems to accept this perspective for a brief while, and subsequently, in line 10, the therapist verbalizes explicitly that she understands the feelings of sadness of the patient (even though the patient has not explicitly admitted to these feelings). The “yes” of the patient in line 11 (see Figure 2) indicates the acceptance of this emotional empathic perspective on herself, which is unusual since she often rejects this emotional side of herself. Thus, the pause in line 12 occurs at a sensitive moment in the interaction allowing both the patient and the therapist to “rest” in this emotion for a brief while. The pause is then followed by a marked in-and-out breath by the therapist that underlines this emotional state even further. The sigh is distinctively expressive as it displays sympathy and understanding of the situation of the patient (see Figure 2). Unlike the previous examples, the embodied action, in this case, is not closely intertwined with verbal actions. It was preceded by a relatively long pause and it is not followed by further verbal actions from the therapist.

As such, the sigh qualifies as an action in its own right to a larger degree than in example 1. The sigh functions as an intelligible part of the interaction, as if it were an utterance without being intertwined with verbal actions. However, the sigh is short lived, lasting only a second, and it is primarily emotional in nature. It carries emotional warmth and expresses an emotion of sympathy, but it is not clear to what degree it requires conscious reflection. It, first and foremost, seems to add extra emotional value to the pause that precedes it, therefore allowing for both the patient and the therapist to feel this emotion a bit deeper. However, the patient does not directly respond to the sigh as one often does to a verbal action, and, as such, the embodied mentalizing action of the therapist is not treated as explicit by the patient. This does not mean that it is a straightforward example of an implicit type of mentalizing. Clearly, it shares characteristics with an explicit way of mentalizing since it stands on its own and is evidently directed at the state of mind of the patient. However, since it is primarily emotional in character and seems to require less conscious reflection, it appears as largely implicit although with certain traits of an explicit dimension.

### Therapeutic Background for the Third Case

The third example involves a woman in her mid-twenties and a female therapist in her mid-thirties (the therapist is not the same as the one in the previous two examples). The patient is suffering from social anxiety and obsessive-compulsive disorder. The patient has previously been in treatment for her anxiety disorders without significant results. A recurrent theme in the therapy sessions work has been her interpersonal relationships and, in particular, her difficulties in expressing her own needs and desires, setting proper boundaries, and breaking a pattern of avoidant behavior.

In this excerpt, the patient is trying to convey to the therapist how she is dealing with her difficulties in expressing what she wants. As part of a new positive development, she has now become more aware of her own needs and begun a process of being more explicit about them in conversation with others, although it is still experienced as being difficult. In the extract below, the patient gives an example of this process.

### Analysis of Example 3

**Danish original version**

1. P: jeg har brug for det det ehr [ja:ahr] det det er jo heller ikke nødvendigt at jeg har brug
2. T: [°°mh°°]
3. for det (.) >det er det< øh [åh] jeg synes det er svært stadigvæk den der^*^jeg har brug for^*^
4. T: [°°mh°°]
5. ^*^jeg har brug for^*^ ja.hh phh: ja
6. P: ja he he he
7. T: bare det at sige det højt herinde [det] jeg kan næsten [mærke det]
8. P: [JA] [det er] ubehageligt]
9. T: ja
10. P: hvor det sådan uhm men men

**English translation**

1. P: I need this that's [yea:hr] it it is not really necessary that I need it (.) >it is < eh [oh] I still think it is
2. T: [°°mh°°]
3.hard this ^*^I need^*^
4. T: [°°mh°°]
5. ^*^I need^*^ yes.hh phh: yes
6. P: yes he he he
7. T: just saying it loud in here [it] I can almost [feel it]
8. P: [YES] [it feels] uncomfortable
9. T: yes
10. P: it's like ehm but but

**Figure 3:**
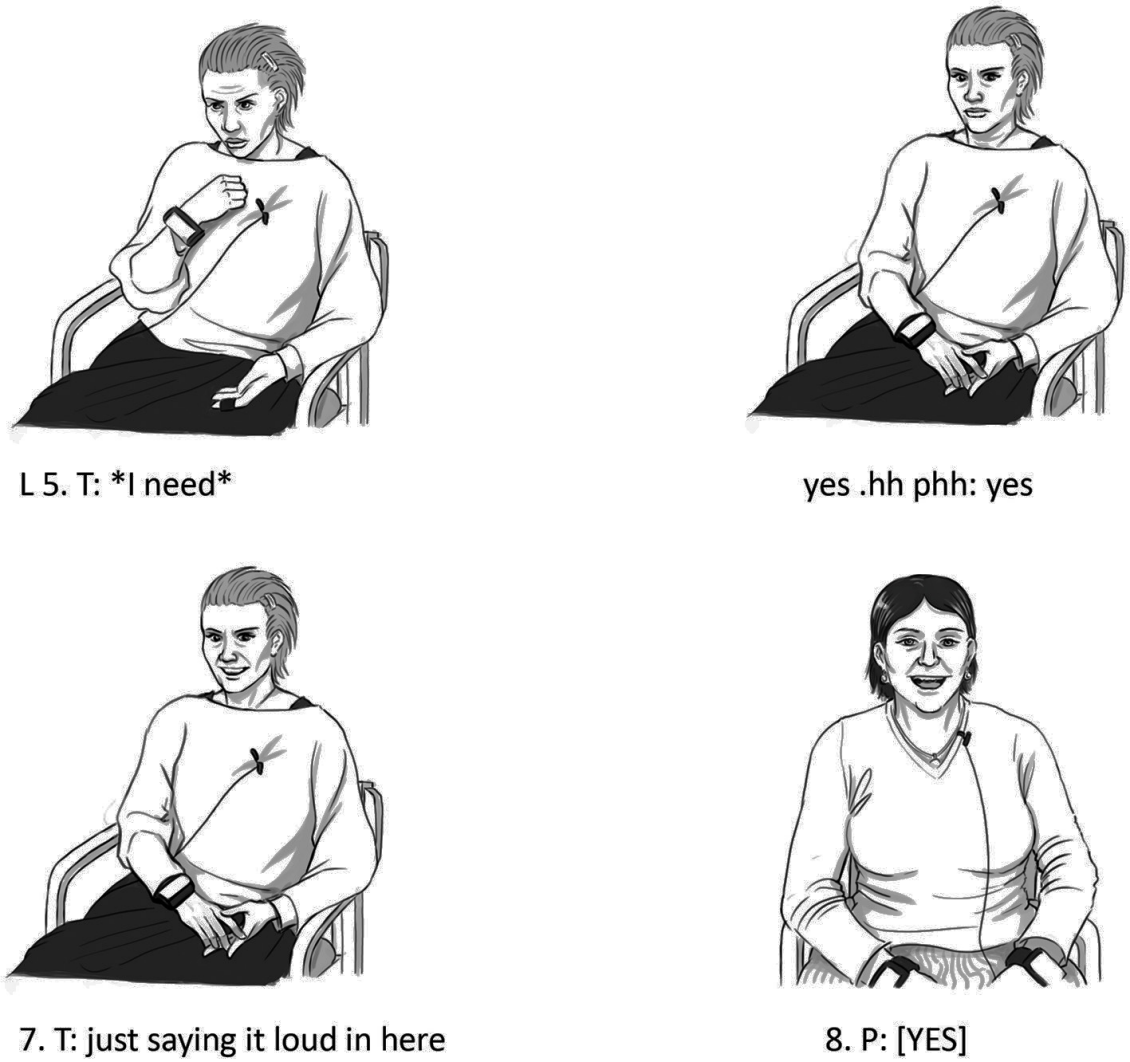
Bodily actions in relation to example 3.

In the first half of the sequence, from lines 1 to 3, the patient is engaged in giving an example of how hard it is for her to express her needs in a straightforward way. The patient uses the phrase “I need this” a few times framed as self-reported speech conveying in a direct manner, a situation in which she has felt the need to express her own wishes in this way. She reports both her words “I need this” as well as her inner thoughts and doubts back and forth about the legitimacy of making such a claim: “ahr it it is not really necessary that I need this (.) it is.” In attending to the description of the patient, the therapist is actively engaged in an embodied pattern of paying attention and giving response. Twice during the description of the patient, she moves her upper body backward while opening her eyes more widely and giving a minimal response in a low voice (lines 2 and 4). This is a typical way of displaying attention and interest during the flow of conversation and, in relation to the theme of this article, a clear-cut type of implicit mentalizing behavior (Fosgerau et al., 2017). However, in line 5, the therapist makes a shift in behavior. First, she repeats the phrase in question “I need this” in an overtly articulated manner while making a pointing gesture directed at herself (see Figure 3) and just afterward, she makes an enlarged in-and-out breath while making a move backward with her upper body (see Figure 3). The marked and overtly enlarged nature of these actions provides them with an affective emphatic quality that suggests that they are carried out from the perspective, not of the therapist, but of the patient. It is, in other words, a marked mirroring displaying an imaginative act of mentalizing as described in the theory section. That is, these embodied verbal and non-verbal actions in combination are directed toward the emotional state of the patient, not only by describing them from the outside but also by attempting to relive them from the perspective of the patient. As such, the therapist adopts an allocentric perspective where she acts and speaks as someone else, in this case, as the patient. As described in the theoretical section, the phenomenon of shifting perspective in this manner by using direct or reported speech is widespread and has been documented in numerous studies (Goffman, 1979; Bakhtin, 1981; Semino and Short, 2004; Prior et al., 2006). However, the phenomenon typically refers to reporting or re-enacting the speech and/or behavior of someone not present in the current conversational situation. Here, the direct speech concerns the interlocutor and is followed by an embodied performance approaching or re-enacting the perspective of the patient. Thus, the embodied actions of the therapist in this part of the sequence are difficult to capture within the standard definition of online implicit mentalization in the literature, such as in this description:

We can respond automatically, mentalizing implicitly. For example, we often respond to others' emotions without thinking about it, nodding sympathetically with a concerned look on our face as we listen to a friend talking about her child's frightening accident. (Allen et al., 2003, p. 2).

In this example, the enlarged and performative character of the actions of the therapist suggests that something more is at stake than an automatic reaction occurring “without thinking about it.” The embodied actions are performative in the sense that they re-enact an emotional reaction seen from the perspective of the patient. Such a shift away from an egocentric perspective is hard to imagine being carried out on an entirely unconscious automatic level.

On the other hand, the embodied actions occur within a very limited time frame (lasting only a few seconds) between two verbal actions at the fast speed of the ongoing conversation, and as such, the embodied actions are closely intertwined with the verbal actions in line 5. Both the verbal repetition just before (“I need”) and the subsequent meta-comment just after the embodied actions, “just saying it loud in here I can almost feel it,” are part of the overall frame of the actions. Thus, the embodied actions in this sequence do not qualify as actions in their own right. That is, they are only intelligible as part of an overall series of verbal actions performed in line 5 by the therapist. Clearly, the embodied actions add extra performative and expressive value to what the therapist is conveying by the use of words. Yet, the embodied actions are primarily meaningful in the context of the verbal actions. Thus, the embodied actions in this sequence illustrate why the distinction between implicit and explicit mentalization is not absolute but can only be understood on a continuum. These actions are neither completely implicit nor completely explicit; rather, it seems reasonable to place them in the middle of the continuum as partly implicit/partly explicit.

In the last example, however, the level of explicit mentalizing is significantly increased.

### Therapeutic Background for the Fourth Case

This example involves a 22-year-old female patient with two young children and the same female therapist as in examples 1 and 3. The patient is diagnosed with borderline personality disorder and generalized anxiety disorder, as well as sub-diagnostic symptoms of obsessive thoughts and panic attacks. The overall topic of this sequence concerns the conflicting emotions of the patient in relation to the hard work of raising two young children. In particular, the example concerns the taboo of admitting that it is sometimes a relief to leave them at the day care in the morning and likewise it may feel exhausting to pick them up in the afternoon. Overall, the patient is detached from her emotions and therefore has difficulties in verbally reflecting on her feelings in a psychologically adequate manner. The therapist has tried to address the problems in a conventional verbal manner but with no significant results. Now, a different strategy from a therapeutic point of view is to approach these problems in a primarily non-verbal embodied manner, rather than only talking about them. The embodied non-verbal approach can be a way to gain access to the detached emotional level of the patient.

### Analysis of Example 4

**Danish original version**

1. T: men hvordan har du egentlig når klokken nærmer sig tre
2. P:.hhh [hhh så jeg]
3. T: [.hhh PHH:: hh]::: hh::.hh phh
4. P::::hh jah sådan ja(h) lige præcis sådan der
5. T: (0.5).hhh PHH:::
6. T: du får brug for virkelig at trække vej[ret dybt ikke]
7. P: [ja hh: ha det] er rigtig det er lige præcis hvordan jeg har det
8. T: hvad griner du af
9. P: det er bare (0.5) jeg kan fortælle præcis hvordan (.) du ved det hvordan jeg har det altså du gør
10. det lige

**English translation**

1. T: but how are you really when it's close to three o'clock
2. P: so yeah.h hhh [.hhh t yes:hh]
3. T: [.hhh PHH:: hh]::: hh::.hh phh
4. P: yeah like that yes heh exactly like this
5. T: (0.5).hhh PHH:::
6. T: you really need to take a deep breath right
7. P: yes hh: he he that's right that is exactly how I feel
9. T: what are you laughing at
10. P: it's just (0.5) you know I can tell exactly how (.) you know it how I feel I mean you just did it

**Figure 4:**
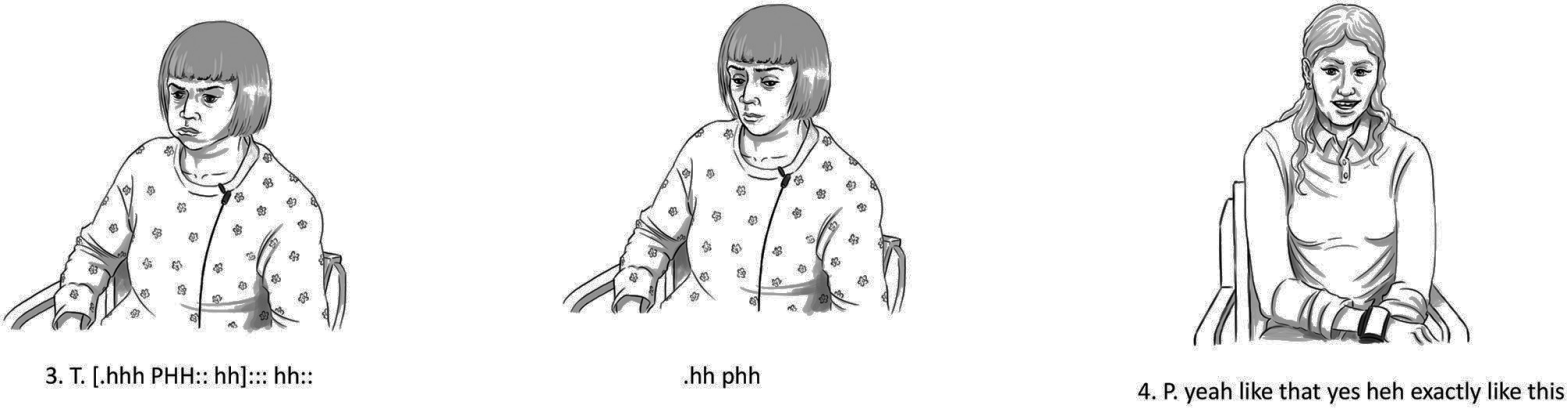
Bodily actions in relation to example 4.

The sequence starts with the therapist in line 1 by asking the patient how she really feels at 3 o'clock in the afternoon, which is the time when she usually goes to pick up her children at the day care. Normally, a direct verbal question, like the one posed by the therapist, requires a verbal answer for a conversation to proceed in an orderly fashion. In this instance, however, the patient does not produce a fully developed verbal answer. She does initiate the beginning of what looks like the beginning of an utterance “so yeah” but then stops and instead, she makes a slightly prolonged in-and-outbreath, transcribed as “.h hhh.” This bodily action is immediately mirrored by the therapist in line 3 in which she, partly in overlap with the turn of the patient, makes two prolonged and clearly marked in-and-outbreaths that are accompanied by an intensified and heightened series of facial expressions in which she, in an overtly exaggerated fashion, breathes in and out (see Figure 4). These embodied actions are clearly visible (and audible) and they are not intertwined with any verbal actions. Rather, they are skillfully coordinated in a way that makes them function as standard contributions to the conversation, but only here, it is not verbal utterances but bodily actions that function as meaningful contributions. They function as independent actions and are also treated as such by the patient through a clear verbal confirmation in line 7: “yeah like that yes heh exactly like this” (see Figure 4).

Furthermore, the actions mark a shift in perspective in a way similar to example 3, only this time the shift away from egocentric perceptive is even more evident. The therapist re-enacts an experience, not from her own life or from her own perspective but an experience originating from the life of the patient and seen from the perspective of the patient, as imagined by the therapist. Furthermore, it is apparent that the exaggerated nature of these bodily actions is used here as “an inter-affective tool” (Colombetti, 2014), that is, instead of talking to the patient about how she feels, the therapist, in effect, shows the patient how she feels. The therapist shows a version of the patient to the patient through her embodied actions, which has a more profound impact than if only verbalized. The complexity and conflicting nature of the experience (feeling exhausted by the prospect of having to pick up her children at the day care) is clearly difficult for the patient to articulate in words, and, as such, it is difficult both to gain access to it on an emotional level and to reflect upon (and perhaps challenge) it on a more conscious level. Thus, it is likely that this shared experience can help to lay the ground for a slight emotional and cognitive change for the patient. In seeing herself from the outside, the patient may be able to see, accept, and understand her own actions better as part of normalizing “forbidden feelings.” As we can see in the excerpt and in Figure 4, the patient is now able to smile and laugh about herself and her own behavior instead of feeling guilty about it.

In relation to the topic of this article, it seems evident that the way in which the therapist manages to address a specific situation and re-enact the anticipated embodied emotional response (from the perspective of the patient) involves a series of complex cognitive and emotional actions that can only be carried out on the level of conscious reflection and affective imagination. Such actions require the therapist to imagine how the patient must feel in a specific situation and, even more importantly, they require a conscious reflection on how to portray and communicate these actions in a way that makes them both recognizable and authentic to the patient. However, this does not necessarily imply that the therapist is conscious of her actions while performing them. But the behavior of the therapist may still be a part of an overall intentional (and affective) stance toward the patient, known as a therapeutic stance (Jørgensen, 2019). This involves a heightened level of affective attention, even though the therapist may not be completely conscious of the particular action. Conscious action is not to be seen as an either-or phenomenon (either completely conscious or not conscious at all); rather, it is a gradual phenomenon with a number of different stages and dimensions (Gibbs, 2005; Gallagher, 2017).

Further, it is important to note that “reflection” is not to be understood as a dis-embodied abstract mental process. Rather, it is a clear case of thinking and acting with and through the body. The bodily actions of the therapist are mindful actions, that is, the mental processes involved in this concrete therapeutic work, namely the perception, the affective attunement, and the performative imagination, are integrated with the in-and-outbreaths and are, as such, inseparable from this embodied activity. According to Merleau-Ponty, it is the phenomenal body: it is a thinking and feeling body, a mentalizing body, that does the in-and-outbreaths, not a body-instrument controlled by the brain.

The therapist clearly succeeds in portraying this situation in a convincing manner since the patient explicates in line 9 how the therapist, in fact, managed to enact (an image of) her own emotional experience: “you know it how I feel I mean you just did it.” The choice of words of the patient is interesting here. The therapist did it, that is, she acted as the patient from an imagined first-person perspective, and that seems to have had a more profound effect on the patient (on a second-person perspective) than a verbal analysis or description.

Summing up, this last example involves a visible and audible embodied action that clearly functions as an intelligible part of the interaction independently of any verbal action. Furthermore, it is evident that it is indeed a mentalizing action directed at the state of the patient, and its performative and imaginative character indicates a certain level of conscious reflection. As such, it is a strong candidate for an instance of explicit mentalizing behavior performed on an embodied level without the use of words.

## Discussion

### Clinical Implications of the Findings

Using multimodal interaction analysis, the article investigates four different empirical examples from psychotherapy of both implicit and explicit mentalizing from the perspective of embodied cognition. This approach made it possible to explore the distinction between implicit and explicit mentalizing as a gradual phenomenon existing on a continuum while at the same time allowing for differentiations between primarily implicit and primarily explicit.

In relation to the clinical implications, we can conclude that (1) when the mentalizing process is studied in detail in empirical data, it is indeed possible to find embodied non-verbal actions constituting explicit mentalization. Further, these investigations add an extra dimension to the notion of “the inquisitive stance,” in which mentalizing activities are seen as “a particular facet of the human imagination” (Fonagy and Bateman, 2019, p. 3). Often, imagination is assumed to rely on language solely (in particular, when being conveyed in conversation). However, in the clinical context of psychotherapy, the analyses pointed out that embodied non-verbal imagination in some cases is at the heart of mentalizing. Examples 3 and 4 both illustrated imaginative acts of re-enactment in which the therapists acted as the patients as an alternative way of conveying understanding and alignment.

(2) A clear clinical advantage of being more aware of embodied non-verbal behavior in therapy is that embodied behaviors, such as gestures, facial expressions, and in-and-outbreaths, can be less intrusive for the patient. Embodied non-verbal behaviors are to a much larger degree open to interpretation, and in that sense, less definite than articulated utterances with a clear and well-defined meaning. In using words, the therapist, in some cases, may risk antagonizing or offending the patient if the topic of conversation has a sensitive character, since the therapist may come across as being too definite or certain in her stance (i.e., the patient may not be ready to hear the words since the therapist is “ahead of” the patient in her interpretation of the situation). In this way, an explicit verbal account or comment may challenge the working alliance, whereas embodied non-verbal actions are more open to interpretation and more likely to display understanding and empathy. In this way, embodied therapeutic actions can work as an invitation for patients to elaborate and to stay curious about their patterns of reactions and the mental states related to them.

(3) The results of the study contain the potential to improve therapeutic treatment as well as further training of therapists. When the therapist re-enacts the emotion which the patient experiences as shameful or in other ways unacceptable, s/he also acts as a role model for acceptance and containment. This intervention can be used early in the treatment and can give the patient a sense of being seen and accepted by the therapist. This can also help to build an effective alliance between the therapist and the patient, which is considered the most important factor of psychotherapy (Lambert and Barley, 2002; Horvath et al., 2011).

(4) Finally, the findings in this study also have clear implications for practices beyond talking therapies, particularly in fields, such as body psychotherapy and dance movement psychotherapy (Payne, 1992, 2017). Within these types of therapy, movement is used to help increase the emotional, cognitive, physical, and social integration of the patients. Inter-relational physical movement is considered beneficial for both physical and mental health and as a tool for stress reduction, prevention of disease, and mood management. At the heart of these forms of therapy is the interrelationship between the mover and the observer, which, on a theoretical level, can be related to the notion of embodied simulation and mirror neurons. Empirical research in these fields (Gallese, 2001, 2005) has documented that through mirror mechanisms on a neurological level, “we can simulate in ourselves the same emotional and somatosensory experiences that we observe in others. This direct, interpersonal route of knowledge allows us to resonate in synchrony with others and makes it possible to share dimensions of experience at a non-conscious level, i.e., that of implicit inter-corporeality” (Payne, 2017, p. 166). The detailed analyses of the present study of marked mirroring involving explicit mentalizing on an embodied non-verbal level can be seen as contributing to both the abovementioned types of psychotherapy as well as to the field of embodied simulation.

### Limitations of the Study

The limitations of the study are first and foremost related to the limited size of this qualitative study. It is based on only four cases, which does not form a basis for more general observations (although it can be used to formulate further hypotheses). Furthermore, the cases were selected on the basis of general research interests within the field of embodied cognition. This means that the study, from the beginning, is shaped by specific research interests, thus making it liable to critique from other research fields with different agendas and methods. Further, as mentioned in the method section, it was the therapists themselves that helped gather the cases and provided inputs for the analyses, which may cause a biased interpretation. However, the analyses were mainly carried out by the first author who is not a therapist and did not have prior knowledge of the patients other than that provided by the therapists.

On a more theoretical note, it is important to clarify that even though the focus of the study is on the process of explicit embodied mentalizing, this type of mentalizing is not necessarily the end-goal. Non-verbal embodied activities can shift on the dimensional scale, ranging from implicit to more explicit, and the potential of these movements often lies in the process of shifting from implicit to explicit. Explicit is not necessarily better, but it is crucial to be able to investigate the movements on the dimensional scale. Following from this, the empirical demonstration of the gradual line between implicit and explicit mentalizing may not appear as a surprising result. It is well-documented that there is a fuzzy line between explicit (conscious) and implicit (unconscious) attention and knowledge (Gibbs, 2005; Gallagher, 2017). Our attention and knowledge move back and forth so to speak. What has previously been explicit (how to ride a bike), is now implicit (know-how). Or reversely, sometimes our habituated know-how (how to open a can) may need to be “upscaled” to a more focused attention (if the can is difficult to open). However, as previously mentioned, in the case of mentalization, this question has often been linked to, and to some degree reinforced by, the distinction between non-verbal and verbal. As previously described, it has been a standard assumption in the literature on mentalization that implicit mentalizing is non-verbal while explicit mentalizing is based on words and articulated reflections. Providing empirical counterexamples against this assumption has been a central contribution of this article.

### Theoretical Perspectives

Lastly, we will discuss the perspectives of this study in relation to contributing to the embodied cognition perspective on explicit and intentional thinking and action.

This study can be seen in continuation of approaches within the fields of intersubjectivity and social cognition in which a more complex model of metacognition has been suggested (Brinck and Liljenfors, 2013; Liljenfors and Lundhl, 2014). Metacognition concerns the ability of the child or adult to mentalize their own cognitions (and as such, it is clearly linked to both the self-other and internal-external dimensions in mentalization theory). Liljenfors and Lundhl (2014) suggest a differentiation between implicit metacognition, perceptual metacognition, and metarepresentational metacognition, where implicit and perceptual metacognition are construed as relying on “heuristics and environmental affordances” (p. 42). Thus, perceptual metacognition does not rely on verbal language or any other kind of metarepresentations (as opposed to so-called metarepresentational metacognition that requires verbal higher-order propositional strategies). Still, it does involve “consciousness and attention-based strategies” (Liljenfors and Lundhl, 2014) along with attention, awareness, and effort, but without being “explicit” in the sense of being verbally expressed. This model is based on the potential metacognition of children but it can also be applied to adults in the sense that “implicit, perceptual, and metarepresentational metacognition coexist in adult subjects, each type contributing in its particular way to the general metacognitive machinery” (Brinck and Liljenfors, 2013, p. 86). In relation to the present study, the concept of *perceptual explicit mentalizing* fits the findings of the analyses perfectly. Liljenfors and Lundhl (2014) suggest, based on theoretical considerations, that it might be beneficial “to divide explicit mentalization into perceptual and metarepresentational—the former relying on attention, awareness, and effort but not on verbal language, and the latter introducing language into the picture?” (p. 42) Based on the analyses in the present study, we are now capable of confirming this theoretical suggestion, even though more research into the matter is still needed.

Finally, it is important to consider the role of the environment in relation to the “mental processes” of imagination and reflection as inherent in the perspective shift or the re-enactment that characterizes example 4. One way to think about this question in a different manner is to relate it to the notion of *affordance space* as proposed by Gallagher (2017). This concept derives from Gibson's notion of affordances (Gibson, 1979), which stresses the idea that the environment often invites, or makes possible, certain types of behavior while excluding others. In social interaction, such as therapy, a vital affordance is the possibility for impulsive action and thought enabled by the interactive environment in the here-and-now of thinking and feeling together. Thus, the argument from an embodied and ecological perspective is that the explicit (reflective and imaginative) part of mentalizing behavior that we saw in example 4 (and in part in example 3) is not just relying on the inner mental properties of the mind of the therapist but is, in equal measures, an emergent property of the therapist–patient-environment. This environment is to a large degree an arranged environment (both in physical and social terms) set out to advance a close, both physical and affective (“mental”), contact between the participants. Thus, the argument from an embodied/ecological perspective would be that this therapeutic environment offers particular types of actions that would be less obvious in other situations (a job interview or a work-related meeting, for instance). To some degree, the opportunity for a shift in perspective is latent as an affordance in the close interpersonal therapist–patient dyad, that is, to imagine the perspective of the other is a way of attuning to each other and the constraints of the environment:

We should think of imagination first as a kind of active engagement with possibilities. One does not need to generate ideas in one's head about these possibilities if one can “*see”* them in the process of interacting with objects and others. Playacting, as a practice of imagination, allows for expansion of a set of affordances—an expansion of the affordance space. (Gallagher, 2017, p. 193–194).

In this light, the explicit dimension in mentalizing behavior is not solely an inner ability or hidden mental process in the head of an individual, but it is more accurately to be understood as an expansion of the local affordance space. It is a skillful re-configuration of the therapist–patient ecology enabled by mentalizing bodies.

## Data Availability Statement

The datasets presented in this article are not readily available due to patient confidentiality and participant privacy and can only be accessed by researchers connected to the project. Requests to access the datasets should be directed to Professor and project manager Sune Vork Steffensen, s.v.steffensen@sdu.dk.

## Ethics Statement

The studies involving human participants were reviewed and approved by South Danish Regional Committee on Health Research Ethics. The patients/participants provided their written informed consent to participate in this study.

## Author Contributions

TJ wrote the entire manuscript including introduction, theory and method, analyses, and discussion. SH provided comments and analytical insights for analyses as well as general comments for the entire manuscript. MK provided general comments for the manuscript. MB provided comments and analytical insights for the analyses as well as general comments for the manuscript. All authors contributed to the article and approved the submitted version.

## Conflict of Interest

The authors declare that the research was conducted in the absence of any commercial or financial relationships that could be construed as a potential conflict of interest.

## Footnotes

^1^For discussions on these and related issues, see Chemero (2011), chap. 2, and Gallagher (2017), chap. 2.
